# Correlations among Psychological Resilience, Self-Efficacy, and Negative Emotion in Acute Myocardial Infarction Patients after Percutaneous Coronary Intervention

**DOI:** 10.3389/fpsyt.2018.00001

**Published:** 2018-01-23

**Authors:** Neng Liu, Shaohui Liu, Nan Yu, Yunhua Peng, Yumei Wen, Jie Tang, Lingyu Kong

**Affiliations:** ^1^Department of Geriatric Cardiology, Xiangya Hospital, Central South University, Changsha, China; ^2^Health Management Center, Xiangya Hospital, Central South University, Changsha, China; ^3^Department of Radiology, Xiangya Hospital, Central South University, Changsha, China

**Keywords:** psychological resilience, self-efficacy, negative emotions, acute myocardial infarction, percutaneous coronary intervention

## Abstract

**Objective:**

We investigated the influencing factors of the psychological resilience and self-efficacy of acute myocardial infarction (AMI) patients after percutaneous coronary intervention (PCI) and the relationships of psychological resilience and self-efficacy with negative emotion.

**Methods:**

Eighty-eight participants were enrolled. Psychological resilience, self-efficacy, and negative emotion were assessed with the Psychological Resilience Scale, Self-Efficacy Scale, Zung Self-Rating Anxiety Scale (SAS), and Zung Self-Rating Depression Scale (SDS), respectively. Furthermore, the relationships of psychological resilience and self-efficacy with negative emotion were investigated.

**Results:**

The average scores of psychological resilience, self-efficacy, anxiety, and depression were 70.08 ± 13.26, 21.56 ± 9.66, 53.68 ± 13.10, and 56.12 ± 12.37, respectively. The incidences of anxiety and depression were 23.90% (21/88) and 28.40% (25/88), respectively. The psychological resilience and self-efficacy scores of AMI patients after PCI varied significantly with age and economic status. SAS scores and SDS scores were significantly negatively correlated with psychological resilience and self-efficacy.

**Conclusion:**

Negative emotions in AMI patients after PCI are closely related to psychological resilience and self-efficacy. Therefore, anxiety and depression could be alleviated by improving the psychological resilience and self-efficacy of patients undergoing PCI, thus improving patients’ quality of life.

## Introduction

In recent years, coronary heart disease has become one of the chronic diseases that pose serious threats to human health, and its incidence has been increasing year by year (1). One new patient is diagnosed with coronary heart disease every 25 s in the United States, and about 34% of patients die in the same year, equivalent to one death per minute (2). Psychological problems in patients with cardiovascular disease have attracted great attention (3). One study found that 19–66% of patients with acute myocardial infarction (AMI) develop anxiety or depression, which in turn increases the mortality of AMI patients (4).

As an important treatment for coronary heart disease, percutaneous coronary intervention (PCI) has a significant effect on improving myocardial ischemia caused by acute or persistent ischemia and hypoxia in coronary arteries (5–7). Since it is an invasive treatment, PCI is a serious stressful event for patients. Several studies have shown that PCI patients have serious psychological problems, including high levels of anxiety and depression, strong disease uncertainty, and low self-evaluation, which can affect the health of patients (8–11).

However, with the rise of positive psychology, the study of mental health problems in AMI patients after PCI is no longer confined to negative psychological factors, but has expanded to positive psychological factors. Psychological resilience and self-efficacy are two hot topics in this area.

Psychological resilience is the ability of individuals to maintain their healthy and orderly development in the face of various unfavorable factors (12–14). Good psychological resilience plays a positive role in promoting disease development and prognosis, can significantly slow the progression of the disease, reduce the body’s inflammatory response to protect the damaged myocardium, and play a protective role in patients with myocardial infarction (15). Studies have shown that for patients with coronary heart disease, the prognosis is better when patients have higher levels of mental flexibility (16). Therefore, improving patients’ psychological resilience can effectively promote their physical and mental health.

The concept of self-efficacy was proposed by Bandura in 1977 on the basis of the surgery-related efficacy expectations (17). Self-efficacy refers to the confidence or belief of an individual in his/her ability to overcome a difficult situation or accomplish a certain behavioral goal, and its main role is achieved through motivational, cognitive, selection, and emotional reaction processes. Studies have shown that the stronger patients’ self-efficacy, the more confident they are in the face of adversity and the fight against their disease, and the better able they are to face the disease with a positive and healthy attitude (18–21).

The present study aimed to investigate the influencing factors of the psychological resilience and self-efficacy of AMI patients after PCI and analyze the relationships of psychological resilience, self-efficacy with negative emotion, which would provide a theoretical reference for clinicians to develop practical measures.

## Materials and Methods

### Participants

Eighty-eight participants were recruited to participate in this study. All research protocols were explained to the participants, and they signed the written consent form approved by the local IRB (Xiangya Hospital of Central South University of Hunan Province, Changsha, China) before any examinations. Patients were randomly selected from the Department of Geriatric Cardiology of Xiangya Hospital, Central South University, Changsha, Hunan Province from March 2017 to November 2017. Inclusion criteria were as follows: age ≥ 18 years; patients with AMI (defined by the presence of typical prolonged chest pain accompanied by serial changes on the ECG and an increase in cTnI above the upper normal range) (22); whether PCI was performed was based on the results of coronary angiography decisions. There were 74 males (84.1%) and 14 females (15.9%), aged 30–82 (62.1 ± 13.1) years.

### Procedures

Participants were inpatients in the Department of Geriatric Cardiology of Xiangya Hospital. The questionnaire was issued on the third day after coronary stent implantation (the day of the coronary stent implantation was the first day). The respondents completed the questionnaire independently under the guidance of unified instruction, and the contents of the questionnaire were in accordance with their own actual situation and self-perception. For patients with special circumstances who could not fill out the forms by themselves, the researchers read the questionnaire out loud sentence by sentence and wrote the answers on behalf of the patients after the patients made their choices.

### Survey Scale

#### Self-compiled Questionnaire of General Situation

The general situation questionnaire included age, sex, education, residence, average monthly family income, payment of medical expenses, other diseases or complications, the duration of coronary heart disease, the number of stent implantations, and the number of stents.

#### Anxiety and Depression Scales

The anxiety level of patients was evaluated by the Zung Self-Rating Anxiety Scale (SAS), consisting of 20 items. A score ≥50 points indicates anxiety; the higher the score, the higher the degree of anxiety. The Cronbach’s α coefficient of the scale is 0.887 and the content validity is 0.910, while the internal consistency of the scale is good. The depression degree of patients was assessed by the Zung Self-Rating Depression Scale (SDS). The total score ≥50 is classified as depression-positive. The higher the score, the higher the degree of depression. The Cronbach’s α coefficient of the scale is 0.885 and the content validity is 0.826, while the internal consistency of the scale is good (23, 24). These two scales can be used in hospitalized patients (25).

#### The Connor–Davidson Resilience Scale

The psychometric questionnaire designed by the American psychologists Connor and Davidson was used to evaluate resilience (26). The reliability and validity of the questionnaire are good, and the internal consistency is 0.91. This questionnaire consists of 25 entries covering three dimensions: toughness (13 items), strength (8), and optimism (4). All entries use a 0-to-4 rating method, 0 meaning not at all 4 meaning almost always the case.

#### Self-efficacy Scale

To measure self-efficacy, we used the general self-efficacy scale (Chinese version) designed by Shi et al. (27), with internal consistency 0.889, test–retest reliability 0.865, and good reliability and validity. The scale involves 10 entries, including the individual’s ability to solve problems, self-confidence to deal with things, achievement of goals, and so on. All entries use a 1-to-4 rating method, and the total score ranges from 10 to 40 points. Self-efficacy is classified into one of three levels according to the score: 31–40 points is considered a high level, 20–30 an average level, and 10–19 a low level. A higher score indicates higher self-efficacy.

### Statistical Analysis

All variables were described with number (*n*), percentage (%), mean (M), SD, and range. Group differences of continuous variables were examined using the independent-samples *t* test or one-way analysis of variance. Correlations among continuous variables were tested using Pearson’s correlation analysis. Statistical analysis was executed by SPSS 22.0 software, and a two-tailed *p* < 0.05 was viewed as statistically significant.

## Results

The average scores of psychological resilience, self-efficacy, anxiety, and depression were 70.08 ± 13.26, 21.56 ± 9.66, 53.68 ± 13.10, and 56.12 ± 12.37 points, respectively. The incidences of anxiety and depression were 23.90% (21/88) and 28.40% (25/88), respectively (Table 1).

**Table 1:** Descriptive statistics for continuous variables.

	Average scores (M ± SD)	Incidence (%)
Psychological resilience	70.08 ± 13.26	–
Self-efficacy	21.56 ± 9.66	–
Self-Rating Anxiety Scale	53.68 ± 13.10	23.9% (21/88)
Self-Rating Depression Scale	56.12 ± 12.37	28.4% (25/88)

Self-Rating Anxiety Scale scores, SDS scores, psychological resilience scores, and self-efficacy scores of AMI patients after PCI varied significantly with age and average monthly income (Table 2).

**Table 2:** Demographic and clinical variables of participants in relation to psychological resilience and self-efficacy scores (scores, M ± SD).

Variable	Psychological resilience (M ± SD)	Self-efficacy (M ± SD)
**Gender**		
Male (*n* = 74)	68.90 ± 13.39	20.57 ± 8.64
Female (*n* = 14)	76.25 ± 12.31	26.75 ± 14.36
	0.320	0.249
**Age (in years)**		
≥60 (*n* = 56)	69.25 ± 15.35	20.06 ± 10.30
<60 (*n* = 32)	71.56 ± 9.06	24.22 ± 8.27
*p*	0.031*	0.026*
**Education**		
Primary school (*n* = 32)	68.22 ± 14.73	23.0 ± 9.24
Junior high school (*n* = 17)	70.40 ± 6.42	14.8 ± 4.65
Senior high school (*n* = 16)	61.40 ± 15.19	19.0 ± 13.28
Junior college and above (*n* = 23)	79.8 ± 9.13	27.17 ± 7.68
*p*	0.235	0.456
**Residence**		
City (*n* = 39)	74.00 ± 12.88	21.36 ± 10.90
Town (*n* = 10)	65.00 ± 23.0	19.67 ± 13.61
Countryside (*n* = 39)	67.54 ± 10.86	22.27 ± 9.66
*p*	0.268	0.834
**Average monthly family income**
≤3,000 (*n* = 52)	63.07 ± 7.92	18.80 ± 9.34
>3,000 (*n* = 36)	80.60 ± 7.91	25.7 ± 9.02
*p*	<0.001*	0.024*
**Payment of medical expenses**		
Health insurance (*n* = 53)	72.67 ± 14.61	20.86 ± 10.59
Rural cooperative medical service (*n* = 35)	66.20 ± 10.44	22.60 ± 8.51
*p*	0.240	0.671
**Co-occurring diseases**		
None (*n* = 35)	68.6 ± 11.19	18.90 ± 9.27
1 (*n* = 42)	70.42 ± 16.22	24.58 ± 9.79
≥2 (*n* = 11)	73.67 ± 8.33	18.33 ± 10.02
*p*	0.850	0.336
**The number of stent implantations**		
1 (*n* = 67)	72.68 ± 13.39	23.26 ± 9.24
≥2 (*n* = 21)	61.83 ± 9.56	16.17 ± 9.74
*p*	0.080	0.119

*p < 0.05 compared within groups.

**Figure 1:**
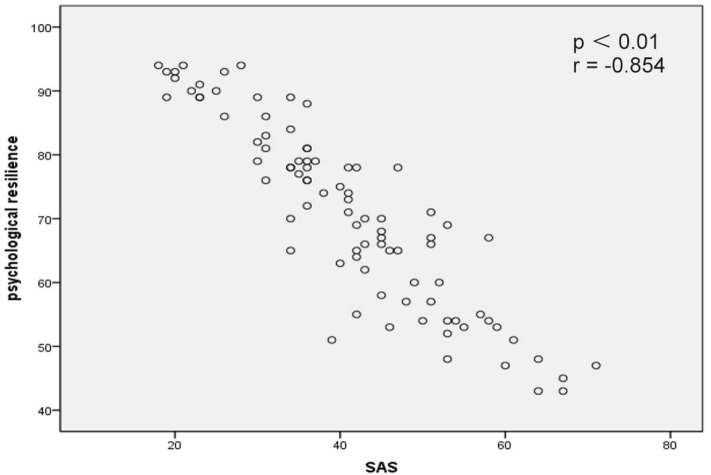
Correlation map between psychological resilience within Self-Rating Anxiety Scale (SAS) scores in acute myocardial infarction patients after percutaneous coronary intervention (*p* < 0.01, *r* = −0.854).

**Figure 2:**
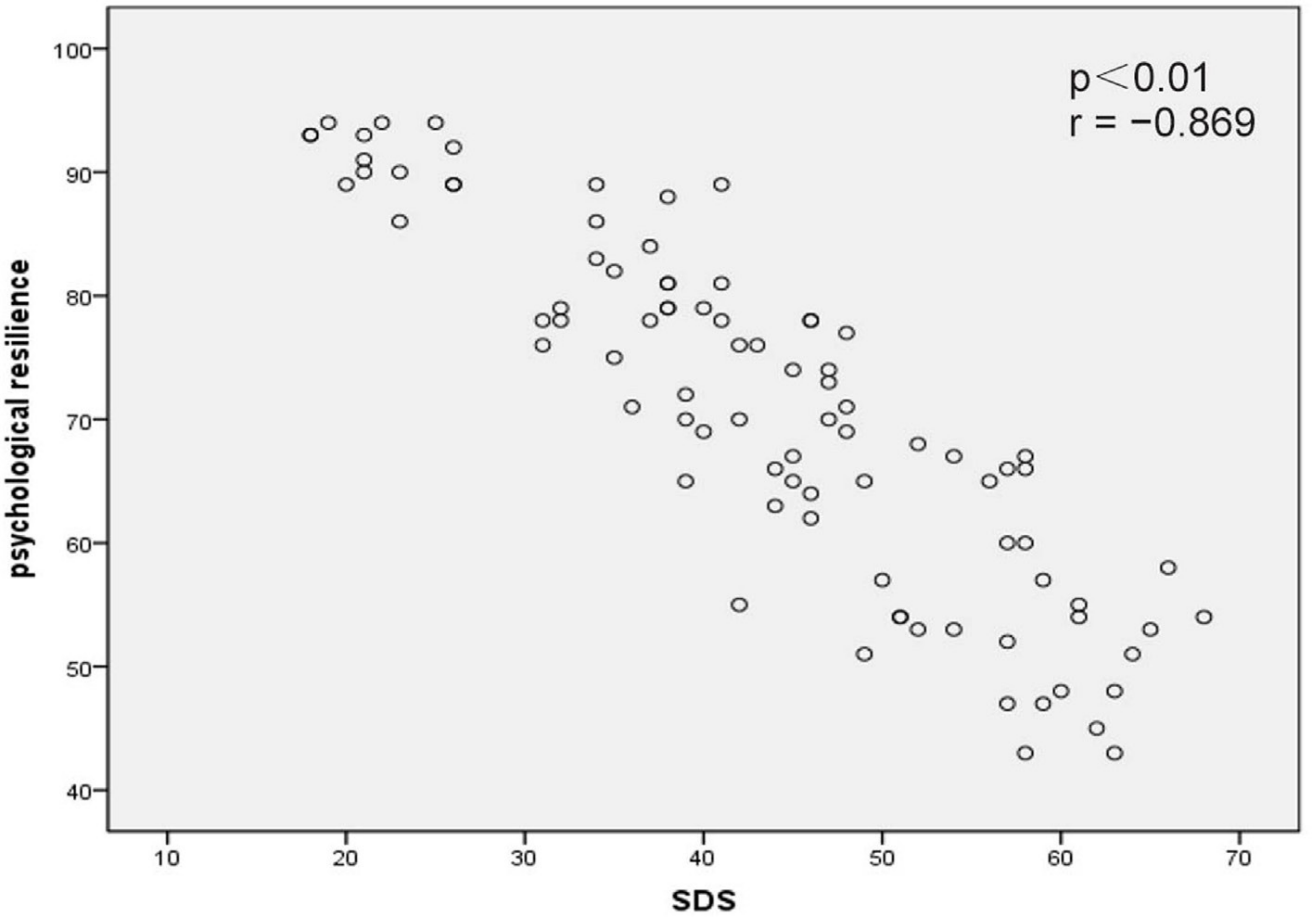
Correlation map between psychological resilience within Self-Rating Depression Scale (SDS) scores in acute myocardial infarction patients after percutaneous coronary intervention (*p* < 0.01, *r* = −0.869).

**Figure 3:**
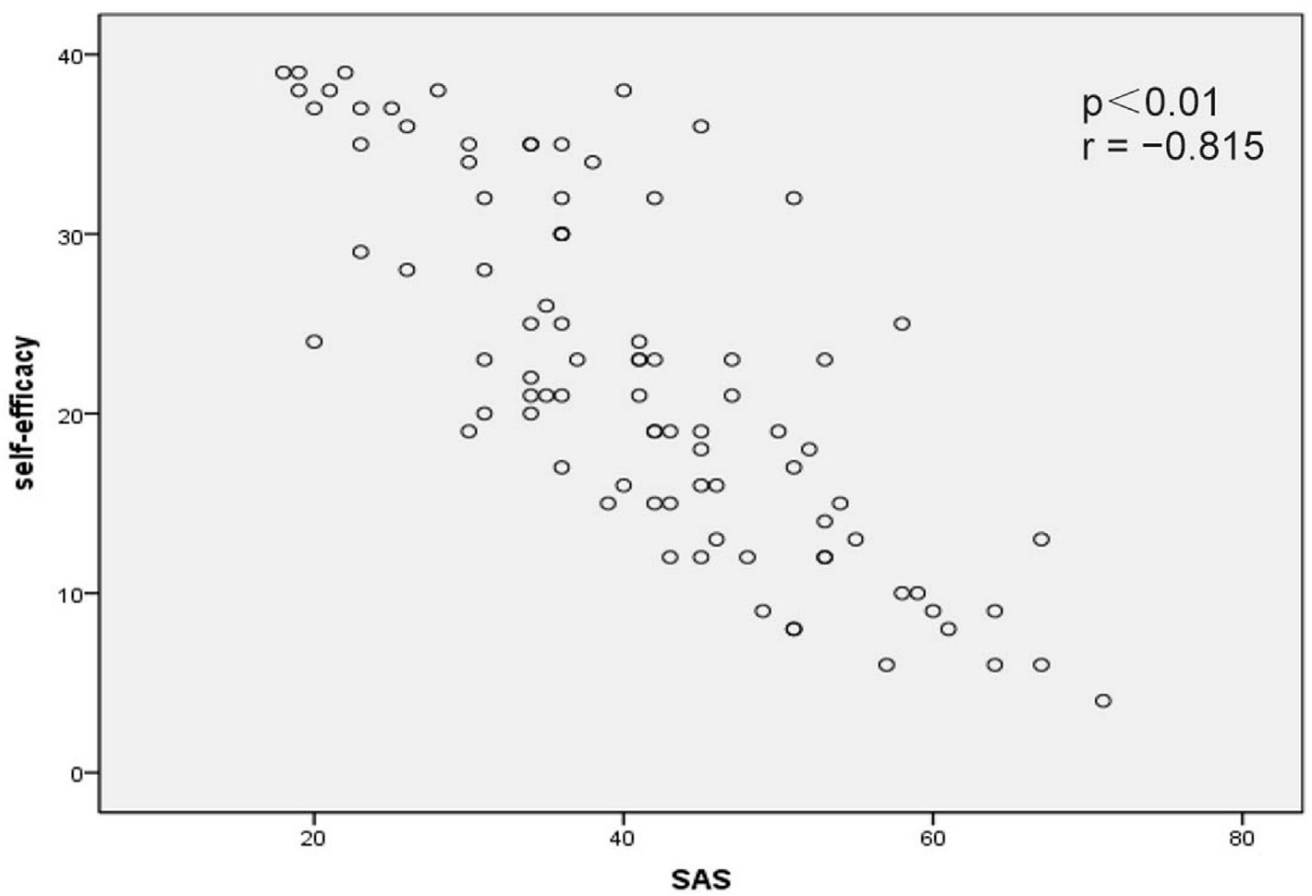
Correlation map between self-efficacy within Self-Rating Anxiety Scale (SAS) scores in acute myocardial infarction patients after percutaneous coronary intervention (*p* < 0.01, *r* = −0.815).

**Figure 4:**
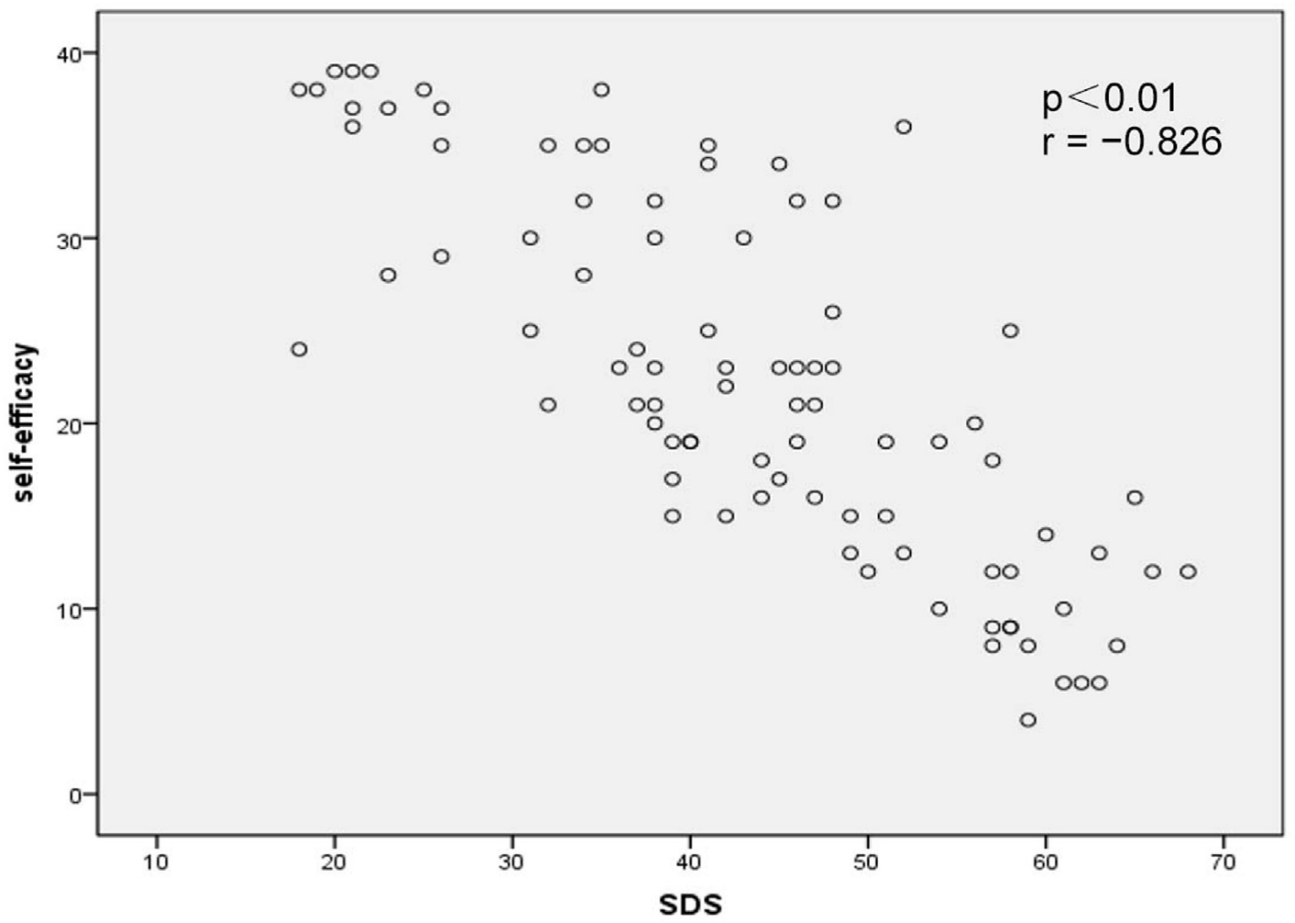
Correlation map between self-efficacy within Self-Rating Depression Scale (SDS) scores in acute myocardial infarction patients after percutaneous coronary intervention (*p* < 0.01, *r* = −0.826).

Correlation analysis revealed that psychological resilience was significantly negatively correlated with SAS scores (*p* < 0.01, *r* = −0.854; Figure 1) and SDS scores (*p* < 0.01, *r* = −0.869; Figure 2). In addition, self-efficacy was significantly negatively correlated with SAS scores (*p* < 0.01, *r* = −0.815; Figure 3) and SDS scores (*p* < 0.01, *r* = −0.826; Figure 4).

## Discussion

In the present study, we recruited 88 AMI patients after PCI to investigate the relationship of psychological resilience and self-efficacy with negative emotion. The results suggested that different ages and different average monthly incomes were the influencing factors of the psychological resilience and self-efficacy of AMI patients after PCI. Specifically, psychological resilience and self-efficacy were negatively correlated with SAS and SDS scores.

As a major source of stress response, AMI has great physiological and psychological impacts on patients, and after PCI, the treatment of AMI brings significant adverse reactions in patients, which will further increase the patients’ psychological burden, potentially leading to PCI-related anxiety or depression. Gu et al. (10) found that negative emotions in patients with AMI peaked on the first day after PCI. The findings of the present study showed that the incidence of SAS in patients with PCI was 23.9% (21/88) and that of SDS was 28.4% (25/88), consistent with the findings of Polikandrioti et al. (28) and Suzuki et al. (29). Although the mechanism of the relationship between negative emotions and cardiovascular disease is not yet clear, anxiety and depression play an important role in the occurrence, development, rehabilitation, and prognosis of coronary heart disease (30), and they are extremely unfavorable to patients’ rehabilitation and prognosis. Some scholars note that as a positive psychology model, psychological resilience plays an important role in eliminating these negative emotions and in the overall care of cardiovascular patients (31).

The study showed that patients after PCI had an average resilience score of 70.08 ± 13.26 points, lower than that of the general population (80.4 ± 12.8) (26), indicating a low level of psychological resilience in patients undergoing PCI. The reason might be that some patients have anxiety, depression, fear, and other psychological problems, thus affecting their adaptation to the disease, resulting in poor psychological resilience. A single intervention study with 226 breast cancer patients found that patients with higher psychological resilience could have a 3-to-5-year increase in survival (32). Lossnitzer et al. (33) also confirmed that the relationship between psychological resilience and the patient’s own psychological variables was close, but found that it was not closely related to the patient’s disease severity, which corroborates our finding that there was no significant correlation between psychological resilience scores and the number of stents used. Therefore, it is necessary for health care workers to communicate with patients and their families and provide support and assistance to patients through concern, affection, and psychological counseling in order to allow patients to share their pain and fear, relieve their pressure, help them understand themselves better, and improve their psychological resilience mechanism against diseases.

Self-efficacy, the confidence and belief of individuals in their ability to effectively implement own actions or overcome difficulties, is the core of self-management. Studies have shown that self-efficacy is conducive to the establishment of a healthy and positive attitude in patients, which helps to improve the patients’ confidence in the treatment of their disease (34). In this study, the average self-efficacy score of patients after PCI was 21.56 ± 9.66 points, suggesting that patients have low self-efficacy after PCI. This might be because under the influence of diseases and serious adverse reactions, the psychological moods of patients change greatly, so that patients in such an environment may lack confidence. Health care workers should attach importance to cultivating and improving patients’ general sense of self-efficacy; changing patients’ attitudes and perceptions of discomfort caused by PCI through various means, such as developing self-care programs and strengthening health education for patients; enhancing patients’ confidence in treatment; and guiding patients to be active in dealing with the adverse effects of disease and PCI in order to maintain a good state of mind during their treatment, thereby improving their quality of life.

This study found significant differences in psychological resilience and self-efficacy scores depending on age and economic status. The psychological resilience and self-efficacy scores of elderly patients were lower than those of middle-aged and young patients, which may be related to their poor psychological endurance and psychological adaptability (35, 36). Self-efficacy also varied with economic status, which may be because patients with poor economic status have heavy financial burdens, which leads to greater psychological stress; patients with poor economic status also generally have poor psychological endurance and are prone to anxiety, depression, and other symptoms during treatment.

Self-Rating Anxiety Scale scores and SDS scores in our study were negatively correlated with psychological resilience and self-efficacy (*p* < 0.05), suggesting that there was a close relationship between negative emotions and psychological resilience and self-efficacy in patients after PCI, which was consistent with studies by Rigby et al. (37), Carvalho et al. (38), and others. Higher anxiety and depression are often accompanied by low psychological resilience and self-efficacy. Patients with higher self-efficacy have stronger confidence in their treatment, higher psychological resilience, lower risk of stress disorder, and lower anxiety or depression. This suggests that clinicians and patients’ families should try to improve patients’ self-efficacy and enhance patients’ confidence in treatment in order to improve the patients’ psychological resilience and improve their mood.

In sum, the negative emotions of AMI patients after PCI were closely related to psychological resilience and self-efficacy. Steps should be taken to help patients with older age and poor financial status to improve their psychological resilience and self-efficacy as well as reduce their anxiety and depression.

A few limitations of this study should be noted. First, the sample size was relatively small. Second, this was a cross-sectional and correlational study, which limits the ability to infer causality. Third, further longitudinal studies should be conducted to replicate and expand on the findings of this study.

## Ethics Statement

All participants were aware of the purpose of the study and signed an informed consent before the study. All research protocols were explained to the participants, and they signed a written consent form approved by the local IRB (Xiangya Hospital of Central South University of Hunan Province, Changsha, China).

## Author Contributions

NL, SL, and LK conceived and designed the experiments. NY, YP, YW, and JT conducted the experiments and collected data. NL and LK analyzed the results and wrote the main manuscript text. All authors reviewed the manuscript.

## Conflict of Interest Statement

The authors declare that the research was conducted in the absence of any commercial or financial relationships that could be construed as a potential conflict of interest.
